# Thriving in the tropics: spatial variation in heat resilience in the early diverging land plant, *Marchantia inflexa*

**DOI:** 10.1093/aobpla/plaf028

**Published:** 2025-06-02

**Authors:** Hansika K Herath, D Nicholas McLetchie

**Affiliations:** Department of Biology, University of Kentucky, 101 T.H. Morgan Building, Lexington, KY 40506-0225, United States; Department of Biology, University of Kentucky, 101 T.H. Morgan Building, Lexington, KY 40506-0225, United States; Plants, Ecosystems & Climate

**Keywords:** *Marchantia inflexa*, heat stress resilience, sex differences, population sex ratios, liverwort, climate change, environmental variation

## Abstract

Increasing frequency and intensity of global warming pose a profound threat to plant species persistence. Most investigations on plants’ resilience to heat events focus on few genotypes of model species. Novel insights into resilience mechanisms will be gained by focusing on natural variation in thermotolerance and its relationship to local-abiotic factors. Additionally, studying species that survived ‘ancient periods’ of high temperatures provides insight into resilience mechanisms. Within a species, we assessed spatial thermotolerance variation, its association with temperature and light, while testing for thermotolerance sex differences and its relationship with population sex ratios. We used *Marchantia inflexa*, a species with unisexual individuals exhibiting spatial variation in physiologies and life histories. To assess field basal thermotolerance (field BT), we examined the efficiency of photosystem II recovery following a heat treatment (53°C for 45 min) in over 200 field-collected plants from seven sites. We further examined whether field BT is linked to initial physiological traits or environmental factors and assessed its potential as a predictor of sex ratios. Following the heat treatment, plants exhibited damage and were still recovering by day ten; recovery was generally higher in road- relative to stream-collected plants with notable variation among sites. Thermotolerance was positively associated with light and tended to be negatively associated with temperature. This light-thermotolerance relationship was more pronounced in males, and thermotolerance differences between females and males tended to be positively related to the proportion of females. The positive light-thermotolerance association suggests that light is a key factor driving heat stress resilience in *M. inflexa*. The light-thermotolerance relationship for males vs. females implies sex-specific strategies for coping with abiotic stress. There were subtle thermotolerance impacts on population sex ratios. These insights broaden the understanding of the thermotolerance diversity present within a species.

## Teaser

An investigation into the heat resilience of natural plant populations of the tropical bryophyte, Marchantia inflexa, revealed microenvironmental factors at each site play a crucial role in shaping plants’ ability to withstand and recover from a high temperature (53 °C wet heat). Among the environmental factors examined, light stood out as a strong driver of recovery after heat stress. Interestingly, male plant recovery showed a stronger relationship compared to female plants.

## Introduction

The increasing frequency and intensity of global heat events pose a profound threat to plant species survival. These high-temperature events disrupt the physiology, morphology, and reproductive success of plants, which could lead to local population declines, and eventually, species extinctions (Panetta et al. 2018; Breshears et al. 2021; McDonald et al. 2023). As inherently stationary organisms, plants are particularly vulnerable to the impacts of heat stress. Addressing plant resilience to heat stress is therefore crucial, aligning with the second goal of the United Nations on sustainable development, which emphasizes sustainable agriculture and food security in the face of climate change (Beddington et al. 2012).

In general, temperatures exceeding ambient temperature by 10–15°C are considered a heat stress or a heat shock (Wahid et al. 2007). Plants exhibit complex and variable responses to heat stress, influenced by the intensity, duration, and frequency of exposure (Distéfano et al. 2025). The resulting responses include (i) basal thermotolerance, which is the inherent ability to withstand heat stress; (ii) thermotolerance to moderately high temperatures, which refers to the capacity to endure prolonged heat exposure; and (iii) acquired thermotolerance, in which plants develop increased resilience to subsequent heat stress following an initial priming event (Wahid et al. 2007; Hasanuzzaman et al. 2013). While some plant species can endure moderate to high temperatures, others find these temperatures lethal, making them heat sensitive (Geange et al. 2021).

Examining intraspecific variation in thermotolerance, including sex differences, offers a strategic opportunity to uncover underlying thermotolerance mechanisms. Understanding these mechanisms can provide valuable insights for enhancing heat resilience in crop plants (Yeh et al. 2012, Zhou et al. 2015). Nevertheless, most research on thermotolerance focuses on a few traditional model and crop species, with the study plants originating from a few genetically unique lines (Zhao et al. 2020; Guerrero et al. 2024). While this approach offers valuable insights into thermotolerance across species, it overlooks the genetic diversity of thermotolerance among and within populations of a single species. In natural environments, species are typically distributed along temperature gradients, where varying selection pressures can lead to diverse thermotolerance responses within the same species.

There are multiple dimensions of intraspecific variation in plant thermotolerance, including temporal, spatial, plastic and genetic. Temporal variation reflects changes in thermotolerance at different time points/growth stages; for instance, younger seedlings often exhibit lower heat tolerance compared to mature plants due to differences in their physiological development (Wahid et al. 2007). Temporal variation can also reflect seasonal changes in thermotolerance (Sastry and Barua 2017; Grossman 2023). Spatial variation, on the other hand, arises when plants from different geographic regions exhibit distinct heat stress responses, driven by regional adaptation to varying temperature regimes (Knight and Ackerly 2002; Guerrero et al. 2024). Temporal and spatial variation can have plastic and genetic components. Plasticity can play a significant role, as plants can adjust their physiology and metabolism in response to short-term or repeated heat exposures, which allows them to enhance their thermotolerance through acclimation (Nicotra et al. 2010; Bita and Gerats 2013). Genetics can also be a critical component, with different genotypes or sexes within the same species displaying diverse levels of thermotolerance, which reflects underlying genetic differences that can be selected for breeding programmes. (Awasthi et al. 2015; Marks et al. 2016). These dimensions are not all exclusive and can collectively contribute to the adaptive capacity of plants to withstand heat stress across different environments. In a comprehensive study of six tropical woody species, Kullberg and Feeley (2024) found that species differed in thermotolerance plasticity across seasons (temporal) and along a thermal gradient (spatial). Our primary objective is to assess variation in thermotolerance across populations of a single species along natural environmental gradients.

Bryophytes (comprising hornworts, liverworts, and mosses), the earliest lineage of land plants, have persisted through evolutionary periods of significantly warmer and more fluctuating temperatures (Gignac 2001; Mertens et al. 2008; Marchetti et al. 2021) by developing essential mechanisms to withstand extreme land environments, while maintaining a simple plant structure (Clark et al. 2023; Guerrero et al. 2024). These plants may still retain ancestral traits and resilient mechanisms, whereas such traits and mechanisms may have been lost or silenced in later evolved taxa (Liu et al. 2004; Oliver et al. 2005; Marchetti et al. 2021). In fact, many bryophytes are tolerant to air temperatures up to 45°C (113°F) when hydrated, with a few having higher heat tolerance above 59°C (138°F) (Balagurova et al. 1996; Eppley et al. 2011). Bryophytes are less morphologically complex relative to vascular plants, with limited water storage and transport, making them prone to rapid dehydration under water scarcity. Thus, while many are desiccation tolerant (Oliver et al. 2020), they cannot rely on evaporative cooling and quickly equilibrate to their air/substrate temperatures (Glime 2017). In fact, while they can tolerate high temperatures when dry, they are temperature sensitive when wet. For example, the desert moss, *Syntricia cannivervis* Mitt., can tolerate 120°C (248°F) when dry (Stark et al. 2009) but will die at 50°C (122°F) when wet (Stark and McLetchie 2006). Investigating how different individuals of these early evolved plant species sense and respond to stress can lead to scientific discoveries that offer novel solutions for developing stress-resilient crops.

This study aims to examine intraspecific variability in field basal thermotolerance (field BT) of the tropical liverwort *Marchantia inflexa* to deepen our understanding of the dynamic nature of plant responses to their local environment. Previous studies on *M. inflexa* identified several traits that differ across habitats and between sexes, including gemma cup production, growth, sex expression (Brzyski et al. 2014), pore density, and photosynthesis (Groen et al. 2010a, 2010b). Additionally, water stress tolerance varied among five stream sites, with females exhibiting greater tolerance than males at the most mesic site, while not different from males at the most exposed site (Marks et al. 2019). Preliminary studies (unpublished) also indicated variations in heat tolerance in *M. inflexa*, further establishing it as a species with spatially dynamic trait variability. This study aims to assess the spatial variation in field BT within natural populations of *M. inflexa*, to associate field BT with initial physiologies and spatial variation in temperature and light. We also investigated if field BT differed by sex and is associated with field population sex ratios which range from all female to all male populations (Fuselier and McLetchie 2004; Brzyski et al. 2018; Marks et al. 2019).

## Materials and methods

### Study organism

**Figure 1.**
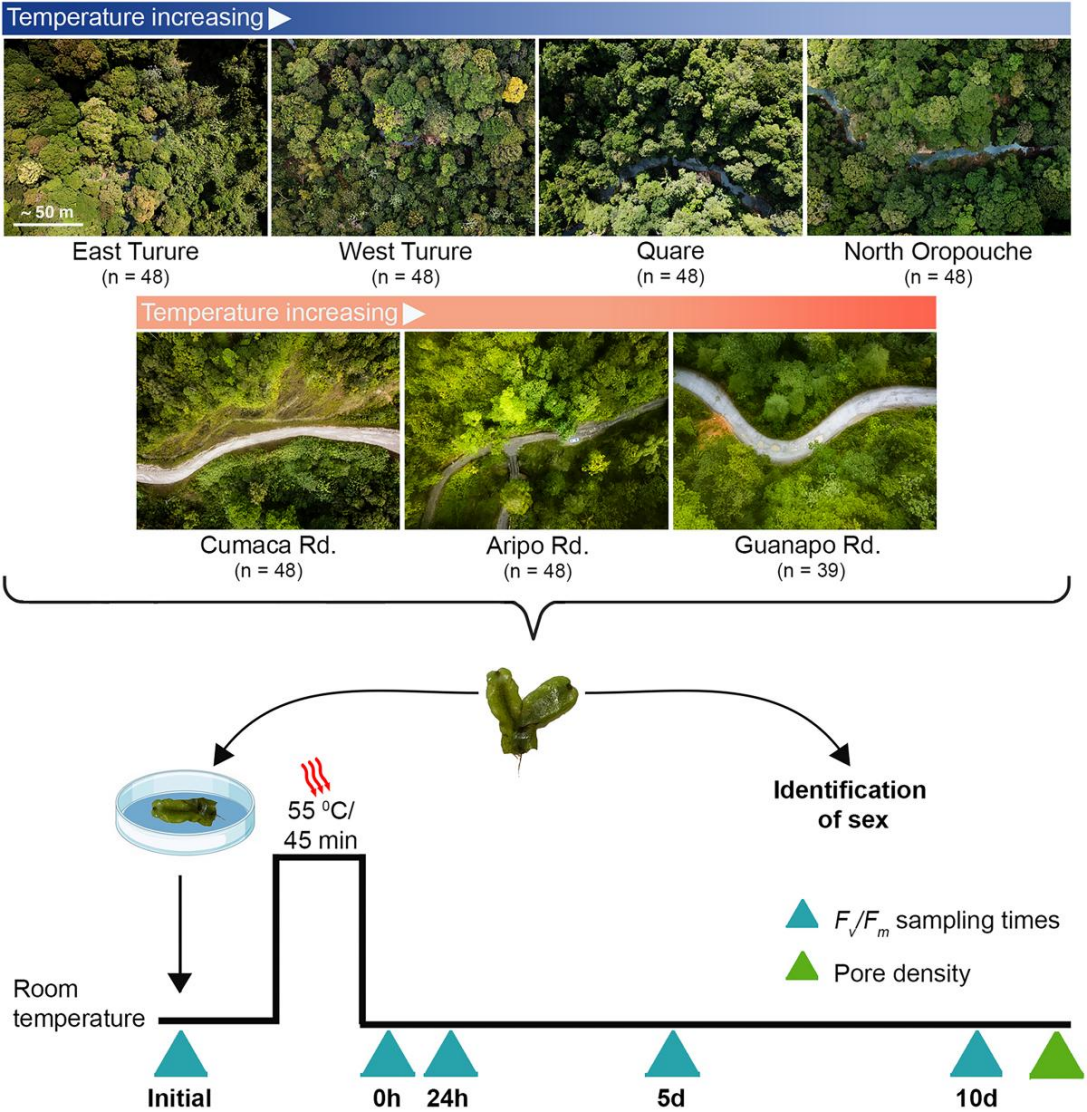
Schematic outline of the experimental design. *Marchantia inflexa* plants were collected from seven field sites that differed in temperature in Trinidad, Republic of Trinidad and Tobago. Aerial photographs of the five sites (in order of coolest to hottest across stream and road habitats and generally colour coded with complementary colours of blue for streams and orange for roads) were taken with a Mavic Pro drone (DJI, Shenzhen China) directly above collection sites at equivalent vertical distances. Areas bordering the streams and roads with an open canopy are shaded blue (streams) or grey (roads) to increase contrast. Plants were subjected to a heat stress event. Plant recovery from the heat stress was quantified by chlorophyll fluorescence to measure the maximum potential quantum efficiency of Photosystem II (*F*_v_*/F*_m_) before, and four times after, the heat event and is expressed as *F*_v_*/F*_m_ recovery (%) (the percent of initial *F*_v_*/F*_m_). Plants were taken to the University of Kentucky for pore counts and a healthy piece of each plant was used for sex identification.

The new world thalloid liverwort *Marchantia inflexa* Nees & Mont. is distributed from northern Venezuela to the southern United States and throughout the Caribbean (Bischler 1984; Marks et al. 2019). *Manchantia inflexa* has unisexual individuals, and they reproduce sexually (via spores), and asexually (via thallus fragments and gemmae). It grows horizontally on the substrate as a bifurcated, flattened photosynthetic thallus, approximately 5 mm wide, forming mats. Additionally, *M. inflexa* has pores on the dorsal surface which are somewhat analogous to stomata in vascular plants for gas exchange (Groen et al. 2010b). However, the pore opening cannot close, unlike the stomatal aperture in vascular plants. Populations are typically found in low light and sheltered habitats, such as along streams. Nevertheless, they can also occur in high light and exposed habitats, such as roadsides (Brzyski et al. 2014), making *M. inflexa* a perfect candidate for this study. Here we used four stream sites (West Turure, East Turure, Quare, and North Oropouche), and three road sites (Cumaca, Guanapo, and Aripo) in Trinidad, the Republic of Trinidad and Tobago (Fig. 1 and Table 1 for coordinates) that were within 14 km of each other. Voucher specimens are deposited at Missouri Botanical Garden (St Louis, MO, USA, specimen numbers M0292113 and M092115) and at the National Herbarium of the Republic of Trinidad and Tobago (St Augustine, Trinidad, specimen number TRIN34616, D.N. McLetchie, collector).

**Table 1.** Site environmental data where *Marchantia inflexa* plants were collected and its sex ratios.

Habitat	Site	Coordinates	Highest temp. (°C)	Mean of highest temp. over 7 days (°C)	Percent canopy openness	PPFD (mol m^−2^ day^−1^)	SR
Stream	East Turure	10°41′22.7″*N* 61°09′37.6″W	26.8	25.8 ± 0.69	11.09 ± 0.14	19.74 ± 3.42	0.67*
	West Turure	10°40′41.6″*N* 61°10′03.0″W	27.2^a^	—	11.04 ± 1.01	9.21 ± 5.64	0.54
	Quare	10°40′29.7″*N* 61°11′47.5″W	28.3	27.2 ± 1.03	12.24 ± 1.07	20.96 ± 3.49	0.32^b^
	North Oropouche	10°40′09.4″*N* 61°08′14.9″W	32.5	28.6 ± 2.80	11.76 ± 1.36	11.54 ± 2.31	0.6
Road	Cumaca	10°40′47.3″*N* 61°09′45.7″W	32.0	29.2 ± 1.90	43.27 ± 7.36	34.26 ± 2.67	0.36^c^
	Aripo	10°40′45.8″*N* 61°13′42.5″W	32.3^a^	—	22.23 ± 4.76	16.25 ± 10.66	0.82***
	Guanapo	10°40′41.3″*N* 61°15′33.2″W	37.9	31.4 ± 4.31	20.33 ± 1.30	27.03 ± 10.20	0.43

Generally, sites with more open canopy had higher light under canopy (PPFD) and were hotter. East Turure stream had one of the lowest percent canopy openness, intermediate PPFD, and was the coolest site, while Guanopo road had an open canopy, high PPFD, and was the hottest site. Sex ratios (SR) are the proportion of females (***P < .0001, *P < .05). Sites are arranged in the order from lowest to highest temperatures. Coordinates were retrieved from a GPSMAP 62sc (Garmin International, Inc, Olathe, Kansas, USA).

^a^Predicted temperatures were used to adjust the positions of West Turure stream and Aripo road on the gradient.

^b^The excluded site from sex ratio analyses due to high mortality.

^c^A tendency (.05 > *P* < .1).

### Site environmental characterization

The streams were perennial with riffles and pools and located within forested areas. Roads were also forested with very low vehicular traffic and thus did not have the constant environmental disturbances that is typically associated with a busy urban road. However, streams and roads exhibit noticeable variation in exposure (e.g. temperature and light), making them fall along a gradient. To characterize the microenvironment at each site and to position them along a temperature gradient, we used data from data loggers and hemispherical canopy photographs. Data loggers were used to record air temperature for five of the seven sites, and data were recorded every 5 min using sensors attached to WatchDog models 450 or 1000 data loggers (Spectrum Technologies Inc., Plainfield, IL, USA). Data were collected at the beginning of the wet season for 7 days in June 2022 (20 June 2022–26 June 2022). Loggers were covered with a radiation shield and placed in the middle of each site at the ground level close to *M. inflexa* plants. Temperature and light data from these five sites were used to predict highest temperatures for the other two sites (see below).

Hemispherical photographs were used to estimate light factors, including percent canopy openness and total photosynthetic photon flux density (PPFD) under the canopy. From each site, three photographs were taken with a Nikon CoolPix 995 camera with a fisheye lens at a height less than 1 m at the beginning, middle, and end of each site. Photographs were analysed with WinSCANOPY software (Regent Instruments Inc., Quebec, Canada), and percent canopy openness and PPFD were estimated for every 7 days from 30 April 2022 to 25 June 2022 (total of 9 days), for each photograph and mean was calculated for each site (see Table 1). A previous study found that percent canopy openness is a measure of exposure and is likely linked to stress (Garner et al. 2017; Marks et al. 2019).

### Collection and sample preparation

Sufficient numbers of bifurcated thallus tips (48 per site, except Guanapo Rd, which had 39, each ∼1 cm long) were collected to maximize the likelihood of sampling individuals of both sexes. Plants were sampled randomly along a linear transect on each site, and at least 1 m apart to ensure obtaining unique genotypes (Brzyski et al. 2018). Upon collection, the isolates were placed in 24-well plates, kept hydrated with stream water, and transported to the research station. At the station, each bifurcated thallus was broken into two tips and one tip was kept hydrated for 24 hours before the initial readings allowing the plants to go through one day-night cycle in room conditions. On the day of the experiment, this tip was placed in a lidded 35/10 mm Petri dish on Whatman no. 1 filter paper with 800 μL of stream water. The other broken tip was transported to the University of Kentucky for sex identification (Marks et al. 2019).

### Testing initial baseline physiologies among individuals

To measure initial quantum efficiency (*F*_v_*/F*_m_) of photosystem II (PS II) of the clipped thalli, an OS5p + modulated chlorophyll fluorometer (Opti-Sciences, Massachusetts, USA) was used. *F*_v_*/F*_m_ is commonly used to assess stress in plants (Krause and Weis 1984; Na et al. 2014; Leon-Garcia and Lasso 2019; Winter at al. 2025). Plants were dark adapted for 20 min before assaying. To account for any possible circadian rhythms, plants were assayed in the morning. Because pores on the thallus allow for transpiration and thus result in evaporative cooling, pore density was used as a physiologically related trait. After the in-situ assessments, thallus tips were transported to the University of Kentucky to determine pore density using a compound microscope.

### Testing field basal thermotolerance among individuals and between the sexes

To detect field BT of *M. inflexa* individuals, thallus fragments from each of the 327 samples were placed in unlidded Petri dishes and subjected to a brief high air temperature event (55°C for 45 min) in the dark. This temperature and duration were selected based on pilot studies and were within the range of other heat stress studies (Wahid et al. 2007). We used 55°C as our assay temperature because we wanted to assess variable responses. Temperatures below 50°C did not result in heat damage in *M. inflexa* as measured by *F*_v_*/F*_m_, while temperatures above 60°C resulted in most plants not recovering (see Supplementary Figure S1). To prevent plants from dehydration, 400 μL of stream water was added after the heat stress. *F*_v_*/F*_m_ was then measured at 0 h, 24 h, 5 days, and 10 days (Fig. 1). Initially the oven was calibrated at 55°C, but with the placement of the Petri dishes with plants inside the oven, the temperature stabilized between the range of 51°C and 53°C. Thus, we refer to the stress temperature as 53°C hereafter. A thermometer was used to monitor the internal oven temperature periodically. Recovery conditions were at ambient light intensity (dim light, <10 μmol m^−1^ s^−2^) and room temperature (27.5°C ± 0.1) with the Petri dishes covered with a transparent lid.

### Statistical analyses

All statistical analyses were performed in JMP Pro, Version 17 (SAS Institute Inc. Cary, NC, USA). We considered *P* < .05 as significant and *P* between .05 and .1 as a tendency that we considered worthy of further discussion because this was a field study and therefore subjected to multiple sources of variation. Physiological data were transformed to improve normality (*F*_v_*/F*_m_ and percent recoveries were arcsine transformed, and pore density was log transformed). We did not apply corrections for multiple statistical tests, because some of our analyses were done to detect potential associations.

#### Analysis of environmental data

Environmental data including mean percent canopy openness, mean estimated PPFD, highest temperatures, and mean highest temperatures for 7 days were summarized by site. To test the relationship between light and temperature, and to use this relationship to predict temperatures of the two sites (West Turure - WT and Aripo - AR) with missing temperature data, we conducted a mixed model repeated measures analysis. We used the recorded temperature and estimated light data—hereafter instantaneous PPFD (µmol m^−2^ s^−1^)—obtained every 15 min from 0750 to 1750 h, ∼40 min after sunrise and before sunset. The dependent variable, temperature was modelled with instantaneous PPFD, daytime block (0750–1250h and 1305–1750 h) and their interaction (instantaneous PPFD * daytime block) specified as fixed effects, time as the repeated measures, and site included as the subject identifier. We analysed streams and roads separately because we felt they might have different light and temperature relationships. We used temperature data from the warmest day (24 June) and instantaneous PPFD data from 25 June where we had estimated PPFD data. Because independent variables were significant in explaining temperature, we used them to predict the temperature of the sites we had instantaneous PPFD but no actual temperatures. The predicted highest temperatures from the two sites, along with those from the other sites, were then used in subsequent analyses to test for patterns associated with temperature.

To quantify the exposure to high temperature at each site, we calculated the number of hours with temperatures exceeding 28°C for the five sites with available data. We used 28°C as a reference because the coolest site did not go above 28°C. To test if the five sites have similar night-time temperatures, the temperature from midnight to 0500 h was averaged for each site for each day for overlapping days, and a one-way ANOVA was performed using the means across the 6 days. Furthermore, percent canopy openness and estimated PPFD were analysed with the effects of sites nested within habitats (hereafter denoted as ‘sites [habitats]’), and habitats.

#### Analysis of initial physiologies

Fifty-six plants died before sex identification, reducing our data set to 271 plants. Then during the heat stress, a monitoring thermometer indicated that the first trial received an air temperature below 50°C (i.e. they were not heat stressed) and these 31 plants (9 from Quare and 22 from Cumaca) were also dropped from the data set (*N* = 240), changing the sample sizes (*n*) in sites to; West Turure = 41, East Turure = 36, Quare = 13, North Oropouche = 45, Cumaca = 23, Aripo = 45, and Guanapo = 37. This data set was then used in the subsequent analyses.

First, we performed a multivariate analysis with initial *F*_v_*/F*_m_, and mean pore density, as the dependent variables with the effects of sites [habitats], habitats, sex, habitat * sex, and site [habitats] * sex interaction. Upon confirming the significance of the whole model, univariate analyses were performed on initial *F*_v_*/F*_m_ and mean pore densities with the same effects. To explore the site [habitats] * sex interaction further, secondary analyses were performed on each habitat separately to test if the sexes differed in the same direction in each site.

#### Analysis of the field basal thermotolerance

Percent recovery was calculated to account for differences in the initial *F*_v_*/F*_m_.


$$(F_{v}/F_{m}{)}_{t}/(F_{v}/F_{m}{)}_{i}\times 100$$

Where ‘*t*’ is the time after the heat event that *F*_v_*/F*_m_ was assayed, and ‘*i*’ is the initial *F*_v_*/F*_m_. To evaluate habitat and sex effects over time and to identify a particular recovery time for further analysis, a repeated measures analysis using multivariate analysis of variance was performed with a full factorial design (time, habitat, site[habitat], and sex). A significant time effect indicates changes in recovery over time. A significant time and main effect interaction indicates habitat, site[habitat], or sex influences the recovery over time. For the subsequent analyses (Student’s *t*-test), we used Day 5 recovery due to its highest *P*-value. To test the factors affecting the percent recovery on Day 5, we used the baseline physiologies (initial *F*_v_*/F*_m_ and mean pore density) as well as the effects of site [habitats], habitat, sex, site [habitats] * sex, and habitat * sex interactions.

#### Analysis of environmental factors with the initial physiologies and field basal thermotolerance

To understand how environmental factors influence female and male initial physiologies and field BT, we performed multiple linear regression analyses by sex. For environmental factors, mean percent canopy openness, PPFD (mol m^−2^ day^−1^), and highest temperatures measured/predicted (on 24 June 2022) from seven sites were used. For initial physiologies, mean initial *F*_v_*/F*_m_ and mean pore density were determined; for field BT, mean percent Day 5 recovery was calculated. Each regression focused on one dependent variable at a time using three environmental factors as predictors.

#### Relationship between environmental factors and physiologies vs. sex ratios

To first identify sex ratio biases, we used a goodness-of-fit test for each site and a heterogeneity test to examine significant variation in sex ratios among sites (Sokal and Rohlf 1995). To test for a relationship between sex ratio and environmental factors, and between sex ratio and physiologies, we used regression analyses. Sex ratios were calculated as female proportions [F/(F + M)]. Due to the high mortality rate (54%), Quare was excluded from all sex ratio analyses. For environmental factors we used three variable: percent canopy openness, PPFD, and highest temperatures.

For physiologies, we used the difference between female and male plants (F-M) for initial *F*_v_*/F*_m_, pore density, and Day 5 recovery. A positive difference for F-M indicates that females have greater values than males. A positive association of the physiological difference with sex ratio indicates that, as females’ physiology level increases relative to males, females are increasing in the population.

## Results

### Site environmental characterization

**Figure 2.**
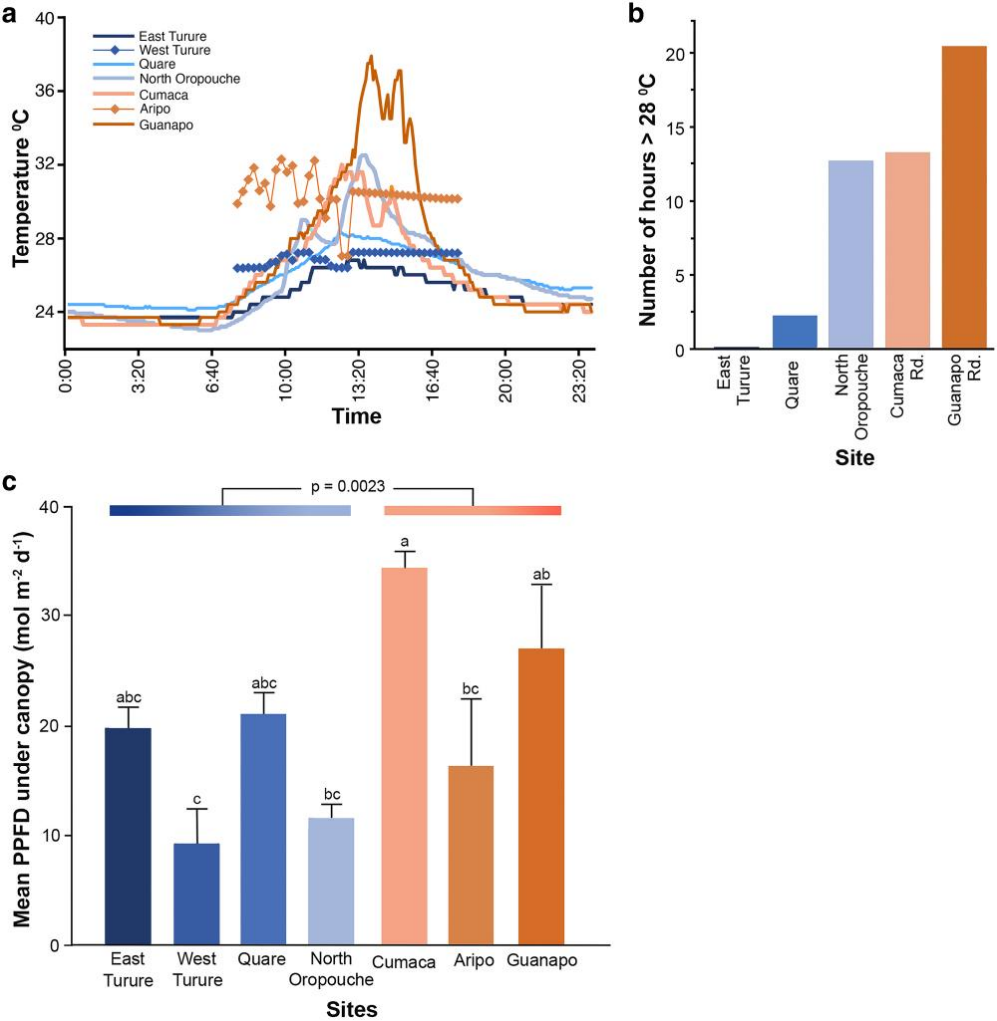
Microenvironmental variation among sites where *Marchantia inflexa* occurred. (a) Temperature fluctuation of Cumaca and Guanapo roads (orange) and North Oropouche, Quare and East Turure streams (blue) for 24 hours (on 24 June 2022) in solid lines, and predicted daytime temperatures for Aripo road (orange solid line with diamonds) and West Turure stream (blue solid blue line with diamonds). Guanapo road had the highest and East Turure had the lowest daytime temperatures, night-time temperatures did not differ among the sites. (b) Cumulative amount of time (in hours) in each site that received temperatures above 28°C from June 20th to 26th, 2022. Aripo and West Turure sites are not included because the data were not collected (c) The sites within habitats differ in total amount of light under canopy (PPFD) reaching *M. inflexa* plants (*P* = 0.0196, *n* = 3 photographs per site). Sites with different letters were significantly different at *P* < 0.05. Streams (blue) had a lower PPFD compared to roads (orange) (*P* = 0.0023). Means are untransformed data and bars are standard errors. Sites are ordered in a gradient of increasing temperatures (see Table 1).

Environmental data were summarized for each site (Table 1). Temperature data were available only in five sites and those data revealed a range in daytime temperatures, with Guanapo road (37.9°C) showing relatively higher temperature compared to East Turure stream (26.8°C) (Fig. 2a). Temperature variation of these three streams and two roads was significantly affected by light, daytime block, and their interaction. Thus, the parameter estimates from these analyses were used to predict the temperatures on 24 June 2022 for West Turure stream and Aripo road using their light data (see Supplementary Table S1). The highest temperatures from these two sites along with the other five sites were then used to order the sites along a temperature gradient.

When characterized for exposure to high temperatures, there was a gradient with East Turure stream receiving 0 hours above 28°C and Guanapo road receiving over 20 h above 28°C over a 6-day period (Fig. 2b). Night-time temperatures were not significantly different among the five sites and were therefore removed from subsequent analyses (Fig. 2a).

The percent canopy openness was significantly higher in road habitats than in stream habitats (*F*_1, 19_ = 127.19, *P* < .0001). Percent canopy openness among sites within habitats were significantly different (*F*_6, 14_ = 16.55, *P* < .0001). The total amount of light under the canopy (PPFD) was significantly higher in the road compared to stream habitats (*F*_1, 19_ = 13.84, *P* = .0023), and was significantly different among sites within habitats (*F*_6, 14_ = 3.93, *P* = .0196) (Fig. 2c).

### Initial baseline physiologies among individuals from different sites

**Figure 3.**
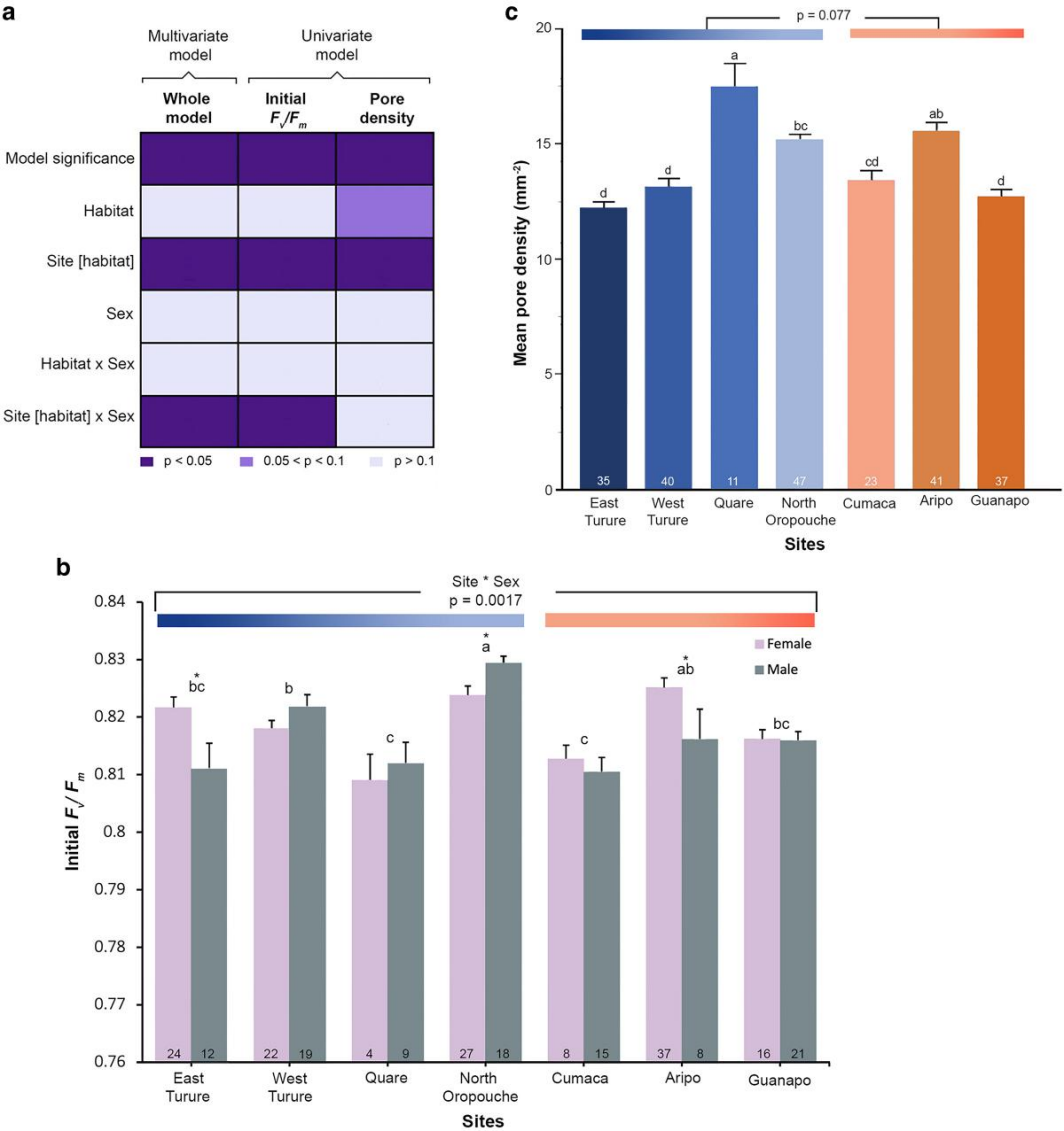
Variation in initial baseline physiologies. (a) Heatmap summarizes the significance of the multivariate and univariate analyses of the dependent variables of initial *F*_v_*/F*_m_, and mean pore density with the effects of habitats, sites within habitats, sex, and their interactions in *Marchantia inflexa*. First row represents the overall model, while rows 2–5 represent the effects, and the columns are the models. The shades of the cells represent that level of significance with the darker shade being *P* < .05 (significant), the intermediate being *P* between.01 and .1 (tendency), and lightest shade *P* > .1 (not significant). (b) Initial *F*_v_*/F*_m_ of *M. inflexa* differs among sites within habitats (*P* < .0001). There was site by sex interaction where in three sites the sexes differed in their initial *F*_v_*/F*_m_ indicated by the asterisks. (c) *M. inflexa* mean pore density differed among sites within habitats (*P* < .0001) and the stream habitat tended to have higher pore density than the road habitat. Sites with different letters are significantly different at *P* < .05. Sites are ordered in a gradient of increasing temperatures. Sample size (*n*) is indicated in the bars, and sample size for pore counts differ from recovery because some thalli died before counts were made.Means are untransformed data and bars are standard errors.

The multivariate analysis, using initial *F*_v_*/F*_m_ and mean pore density as dependent variables was significant (Wilks’ Lambda < 0.0001). While the main effects of habitat, sex and their interaction (habitat * sex) were not significant, main effect of site within habitats (*F*_10, 432_ = 13.80, *P* < .0001), and the site within habitats * sex interaction (*F*_10, 432_ = 2.24, *P* < .015) were significant (Fig. 3a). Partial correlation between initial *F*_v_*/F*_m_ and mean pore density was not significant.

For the univariate analysis of initial *F*_v_*/F*_m_, habitats, sex, and their interaction were not significantly different. However, sites within habitats were different (*F*_5, 226_ = 10.08, *P* < .0001). There was an interaction effect between sites within habitats and sex (*F*_5, 226_ = 3.99, *P* = .0017), where within stream sites female plants were significantly higher in their initial *F*_v_*/F*_m_ compared to male plants in East Turure, while the pattern was reversed in North Oropouche. In only one road site, the sexes differed, with females having higher initial *F*_v_*/F*_m_ than males (Fig. 3b).

For the univariate analysis of pore density, the main effect of sex was not significant. Stream plants tended to have higher pore densities than road plants (*F*_1, 217_ = 3.15, *P* = .077). The interaction between sex and habitat was not significant. Mean pore density was significantly different among sites within habitats (*F*_5, 217_ = 17.18, *P* < .0001) (Fig. 3c). Because there was a tendency for habitats to be different, we focused on the site differences within roads and streams, using site, sex, and their interaction as effects. For both roads and streams, sites were found to be significantly different (*F*_2, 98_ = 9.06, *P* = .0002 and *F*_3, 129_ = 24.83, *P* < .0001, respectively). Overall, sex and site * sex interactions were not significantly different in roads and streams (analyses not shown).

### Field basal thermotolerance

**Figure 4.**
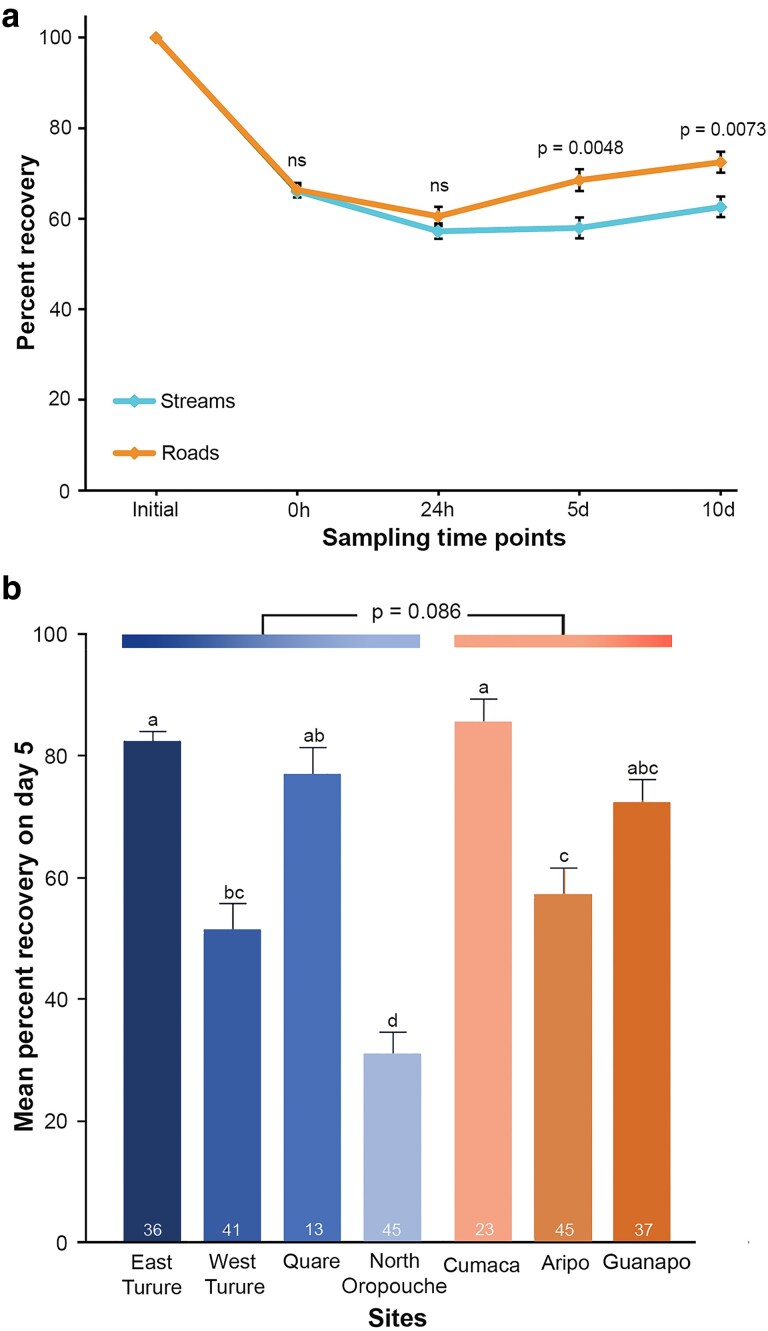
Variations in field basal thermotolerance (field BT). (a) Recovery percentages of stream- and road-collected *Marchantia inflexa* plants over a 10-day period after heat stress (53°C for 45 min). Sample sizes for each habitat were roads = 105 and streams = 139. *P*-values represent analyses of each time point separately, comparing the two habitats using a Student *t*-test. (b) Recovery percentages of *M. inflexa* plants on day five. Recovery calculated by sites nested within stream and road habitats. Sites with different letters are significantly different at *P* < 0.05. Means are untransformed data and bars are standard errors.

Results of the time series analysis indicated a significant overall time effect, denoting an increase in plant recovery over time after heat damage (*F*_3, 224_ = 94.45, *P* < .0001). Overall, heat stress recovery did not differ significantly between the habitats and the sexes, but there was an overall site effect within habitats (*F*_5, 226_ = 25.49, *P* < .0001). However, over time road plants recovered more relative to stream plants (habitat * time interaction, *F*_3, 224_ = 5.41, *P* < .0013). Male and female plant recovery over time (sex * time interaction) and the interaction between habitats and sex over time (time * habitat * sex interaction) were not significant. To further explore the time * habitat interaction, each time point was analysed separately for a habitat effect using a Student’s *t*-test (Fig. 4a). There was no significant difference in the recovery between stream and road plants right after (0 h) and 24 h after (24 h) the heat stress. However, road plants had a significantly higher recovery than stream plants on Day 5 (5d) (*F*_1, 277_ = 8.1, *P* = .0048) and on Day 10 (10d) (*F*_1, 277_ = 7.3, *P* = .0073). Examination of sites within habitats revealed distinct differences in recovery among sites (*F*_6, 272_ = 23.107, *P* < .0001) (Fig. 4b).

Day 5 recovery tended to be higher in road plants relative to stream plants (*F*_1, 229_ = 2.97, *P* = .086), but some road and stream sites overlap in recovery. For example, Aripo Road plants were less resilient than East Turure and Quare stream plants (Fig. 4b). Overall, sex was not different in Day 5 recovery, while sites within habitats were significantly different in Day 5 recovery (*F*_5, 226_ = 18.91, *P* < .0001) (Fig. 4b). All interaction effects, habitat * sex, and site within habitats * sex were not significant. Additionally, Day 5 recovery was not significantly associated with initial *F*_v_*/F*_m_ and tended to be positively associated with mean pore density (*F*_1, 230_ = 3.33, *P* = .069).

### Environmental factors with the initial physiologies and field basal thermotolerance

We first examined how environmental factors relate to initial physiologies by sex. In females, mean initial *F*_v_*/F*_m_ was not significantly associated with canopy openness, PPFD or highest temperatures. In males, initial *F*_v_*/F*_m_ was likewise not significantly associated with canopy openness or highest temperatures; however, initial *F*_v_*/F*_m_ exhibited a negative tendency with PPFD (*F*_1, 3_ = 6.735, *P* = .081). Further, mean pore density was not significantly associated with percent canopy openness, PPFD or highest temperatures for both females and males.

**Figure 5.**
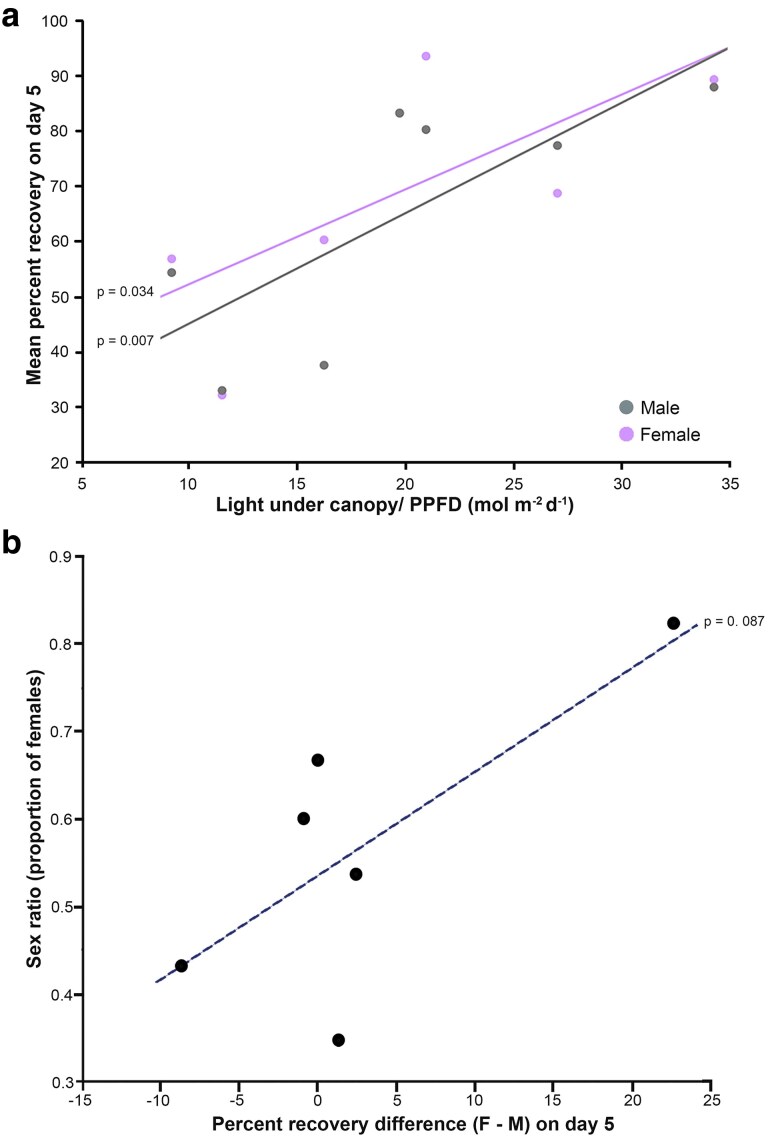
(a) As estimated light under canopy (PPFD) increased, percent recovery of *Marchantia inflexa* at day five after heat stress increased across all seven sites, for both sexes, with females (lilac dash line and markers) being significant, and males (grey solid line and markers) being strongly significant. One lilac marker is not visible because a grey marker is on the top of it. (b) As the percent recovery difference between females and males increases, the sex ratio (proportion of females) of *M. inflexa* tends to increase across six sites. Quare river was dropped due to high mortality after collection which would have affected the estimated sex ratio (see Table 1 for sex ratios).

We then assessed the influence of environmental factors on field BT by sex. In females, mean percent recovery by Day 5 was not significantly associated with the percent canopy openness but showed a negative tendency with highest temperatures (*F*_1, 3_ = 6.686, *P* = .081), and exhibited a significant positive relationship with PPFD (*F*_1, 3_ = 13.208, *P* = .034) (Fig. 5a). In males, recovery tended to decline with both increasing percent canopy openness and highest temperatures (*F*_1, 3_ = 7.780, *P* = .069 and *F*_1, 3_ = 9.844, *P* = .052, respectively), and exhibited a strong positive relationship with PPFD (*F*_1, 3_ = 44.080, *P* = .007) (Fig. 5a).

### Relationship between environmental factors and physiologies vs. sex ratios

The Quare stream site was eliminated from the analysis due to high mortality (54%) and the sex of these plants was unknown. Combined across sites, the sex ratio was female biased (proportion of females = 0.57, G = 4.9, df = 1, *P* = .02). Sex ratio varied across sites (Heterogeneity G test = 25.9, *P* < .0001) with the proportion of females ranging from 0.35 (Cumaca road) to 0.82 (Aripo road) (Table 1). The associations between sex ratio vs canopy openness, PPFD, and highest temperatures were not significant. The associations of the differences in initial *F*_v_*/F*_m_ and pore density between F-M and the sex ratios were not significant. However, differences in Day 5 percent recovery between F-M and the sex ratio revealed a positive trend (*F*_1, 5_ = 5.093, *P* = .087). That is, there was a positive association between relative female recovery (F-M) and the proportion of females in the population (Fig. 5b).

## Discussion

**Figure 6.**
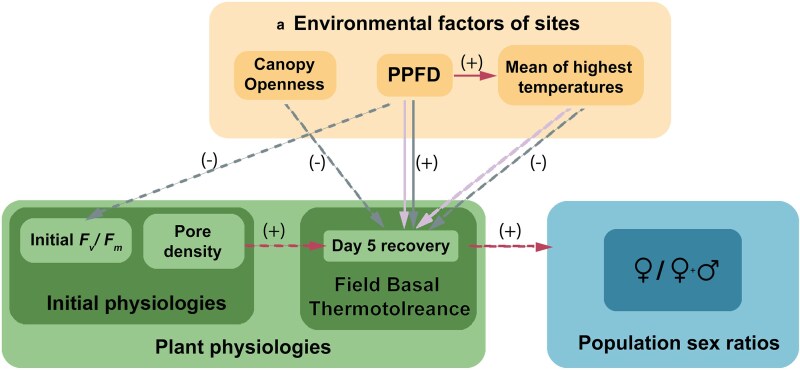
Summary of results. Spatial variation occurred in environmental factors, physiological traits and population sex ratios of *Marchantia inflexa*. The presence of a line indicates a significant association (solid line) or tendency (dash line) between the two variables. Direction of the arrow represents a possible causation relationship. + is a positive association and − is a negative association. Lilac arrows represents female relationships, grey arrows represents male relationships and red arrows represents overall relationships.

We detected spatial variation in physiological traits in *Marchantia inflexa* and found relationships between trait variation with environmental factors, indicating the potential role the local abiotic conditions play in shaping physiological responses. Thermotolerance (as indicated by *F*_v_*/F*_m_ recovery) was significantly increased with daily light exposure while tending to decrease with temperature for both females and males. This suggests light availability, more than temperature, is a factor that can drive heat resilience. Notably, this light-thermotolerance relationship was a stronger and more consistent response in males relative to females, pointing to sex-specific divergent strategies for coping with environmental stress. There were subtle indications that thermotolerance impacts population sex ratios (Fig. 6). These insights broaden the understanding of the diversity of thermotolerant genotypes present within a single species.

### Spatial variation in initial physiologies

The initial physiological traits (*F*_v_*/F*_m_ and pore density) of *M. inflexa* varied significantly across sites within road and stream habitats. These physiological variations result from plastic, genetic, or a combination of both effects emphasizing the need for additional controlled studies to disentangle these effects. Previous research showed genetic differentiation (using microsatellites) between plants originating from roads versus streams (Brzyski et al. 2014), and significant variations among stream sites occur (unpublished data). Moreover, differences in initial photosynthetic efficiencies due to site and sex interactions suggest a complex interplay, where males and females exhibit distinct physiological strategies (e.g. Marks et al. 2019, males vary across two different sites while females do not vary), and these might be linked to the life history variations found in previous studies (McLetchie and Puterbaugh 2000; Groen et al. 2010a; Brzyski et al. 2014). Another recent example from Kullberg and Feely (2024) found spatial and temporal variation among six tropical tree species.

### *Marchantia inflexa* has a high field basal thermotolerance that spatially varies

Plants had not completely recovered by Day 10, and this lack of full recovery was not surprising as similar patterns have been observed in more controlled studies (unpublished data). We speculate this is due to the differing conditions of the recovery environment (warmer in this case). Although full recovery was still incomplete by Day 10, the recovery measurements ceased due to logistical constraints. Nonetheless, plant recovery remained on a positive trajectory. Temperatures above 50°C are often lethal in plants, and most studies on vascular plants use air temperatures up to 50°C to explore the upper limits of heat tolerance (Yeh et al. 2012). For instance, *Arabidopsis* shows significant physiological damage at temperatures exceeding 45°C (Queitsch et al. 2000; Yeh et al. 2012; Hasanuzzaman et al. 2013). In bryophytes, wet heat testing is typically at around 40°C, while temperatures ranging from 42 to 51°C are considered lethal (Alpert 2000; Glime 2017). However, research on the liverwort *Riccia* assessed wet heat tolerance at 50°C (Volk 1984, as cited in Glime 2017). Our pilot studies on *M. inflexa* revealed no significant reduction in *F*_v_*/F*_m_ below 50°C wet heat (see Supplementary Figure S1). Thus, although uncommon in heat stress studies in plants, we used 53°C. Survival at 53°C suggests that *M. inflexa* possesses robust thermotolerance mechanisms. We propose that the mechanisms in *M. inflexa* involve intricate regulatory networks and cellular repair pathways to protect cells from damage and maintain functions under stress as in *M. polymorpha* (Marchetti et al. 2021). This robust thermotolerance positions *M. inflexa* as a promising model for studying mechanisms to improve crop resilience to heat stress.

Consistent with our finding on initial physiological traits, we observed significant variations in field BT among plants from road and stream sites but found no site by sex interaction. Like the initial physiologies, variation in field BT likely has plastic, genetic, or a combination of both effects where controlled studies are needed to disentangle these effects.

### Microenvironment and field basal thermotolerance

Associations of the stream and river sites with their respective environmental data provide insight into plausible causal links between specific environmental factors and field BT. As expected, stream sites were cooler and more sheltered than road sites, and there were overlaps with some roads being more sheltered than some streams. This overlapping is not surprising because all roads traverse forested areas and some streams had very open canopies. Further, in one site (Guanapo road), daytime temperatures were over 37°C for 15 to 30 min and increasing temperatures during the day were positively associated with light (PPFD).

The local spatial variation in thermotolerance and environmental factors reported here for *M. inflexa* is reflected at the global scale in thermotolerance and climate found in *M. polymorpha* L. A recent study using a few individuals found that plants collected from Australia were more heat stress tolerant than plants collected from Japan and the United Kingdom, where the temperature regimes are thought to be milder than in Australia (Guerrero et al. 2024). Interestingly, in our study, higher thermotolerance was more strongly associated with higher light than temperature variation, suggesting that light might be playing a more pivotal role than temperature in heat resilience.

The light and thermotolerance association is potentially influenced by two factors: pigments and temperature-sensitive photosensors. Light enhances thermotolerance through antioxidative mechanisms using the carotenoids of the xanthophyll cycle where violaxanthin converts to antheraxanthin then to zeaxanthin. In shade plants, the lower abundance of carotenoids (Slot et al. 2019), may explain the positive relationship we observed between higher light and increased thermotolerance in *M. inflexa*. However, this relationship cannot be solely due to the xanthophyll cycle, as the plants were collected and maintained under uniform conditions at the research station for a full day prior to heat stress, resetting the xanthophyll cycle. Temperature-sensitive photosensors also play a role. For instance, phototropins (a blue light receptor) in *M. polymorpha* not only control chloroplast positioning but also function as a temperature sensor (Fujii et al. 2017). Similarly, phytochrome B in *Arabidopsis* integrates light and temperature signals to mediate stress responses (Jung et al. 2016; Legris et al. 2016; Hayes et al. 2021). However, in contrast to *Arabidopsis*, where phototropins regulate stomatal movement and temperature-driven responses, *Marchantia* lacks functional stomata, and phototropins are inactivated at high temperatures, leaving their role in thermotolerance unclear (Marchetti et al. 2021). Future studies in controlled environments are essential to uncover the genetic basis of thermotolerance in *M. inflexa* and to better understand the interplay between light and temperature stress. High light levels might be an important stimulus for inducing other stress tolerance responses. For example, high light as indicated by PPFD, red/far-red ratio, and canopy openness were associated with a greater difference in desiccation tolerance between the sexes in the leafy liverwort *Plagiochila porelloides* (Torr. ex Nees) Lindenb. (Silva-e-Costa et al. 2022). Although ultraviolet light resulted in an increase in desiccation tolerance in the *Syntrichia caninervis* (Ekwealor et al. 2021). Increasing light is linked to increasing temperature and decreasing moisture; therefore, the advantages of organisms for using light as a selective agent for water and heat stress tolerance is a reasonable expectation.

### Sex-specific responses to field basal thermotolerance

Although we found no overall sex effects or interactions involving sex on field BT, we detected a sex differential response associated with light. The lack of a general sex response for thermotolerance was surprising in *M. inflexa* because previous studies using plants from many of the same sites found variation in water stress tolerance where females were sometimes more tolerant than males (Marks et al. 2016; Marks et al. 2019). These results are consistent with both stressors having distinct mechanisms in other plants (Rizhsky et al. 2004). Generally, females are expected to allocate more resources to sexual reproduction than males (but see Delph 1999 on wind-pollinated plants) and this allocation is associated with females being less stress tolerant relative to males (Leigh and Nicotra 2003; Bañuelos et al. 2004). Nevertheless, there is no consensus on which sex is more stress tolerant in seed plants, where the sexual dimorphic tolerant pattern is absent in some species or is stress specific (Juvany and Munné-Bosch 2015; Retuerto et al. 2018; Liu et al. 2021). In the few studies on bryophytes, females were found to be more water stress tolerant, or had responses that indicated more water stress tolerance, than males (Newton 1972; Stieha et al. 2014; Marks et al. 2016; Slate et al. 2017; Silva-e-Costa et al. 2022; but see Stark et al. 2005 for a lack of a sex difference in desiccation tolerance). Interestingly, in *M. polymorpha,* the male accession sensed heat (at 27°C) and drought stress faster than the female accession (Guerrero et al. 2024). Although the female had lower *F*_v_*/F*_m_ than the male accession at two stress times, the association of response rate and recovery is not known because recovery was not explicitly tested.

Our exploration of the occurrence of sex-specific physiologies in this current study detected tendencies/significance for an association between PPFD (to a lesser extent temperature and canopy openness) and physiology in males more than in females. Greater association of traits with environmental factors in males relative to females were previously found between canopy openness and edge pore densities (Groen et al. 2010a). Further, dehydration tolerance did not differ in females across varying canopy exposures but differed in males (Marks et al. 2019). We speculate that, relative to females, males track the environment more due to greater plasticity or genetic differentiation. That is, males might have a greater range of fitness optima than females. For example, females must be above a threshold size to mature offspring, but males can be smaller or similar in size to females and still be able to produce gametes to fertilize females. An analogy can be found in some animal species where males have more than one mating type and females have one (Godin 1995; Lank et al. 1995, Cook et al 1997; Taru et al. 2002). Further research is needed to test this speculation.

### Sex ratios and field basal thermotolerance

Many bryophyte population sex ratios are female biased but unlike many seed plant studies, an association between sex ratios with an environmental gradient has not been evident (but see Castetter et al. 2019) in bryophytes. We did not find any evidence of an association between sex ratio and the environmental gradients of light and temperature. Intriguingly, as male thermotolerance decreases relative to female thermotolerance there tends to be an increase in female-biased sex ratios. As noted above, male thermotolerance was more positively associated with light than females, and this could contribute to population sex ratios. However, sex ratios are not associated with light which might be due to other factors that affect female and male demography (e.g. survival and asexual reproduction), coupled with the low sample size (*n* = 6 sites) in the analyses. Further, if increasing heat events affect both sexes differently, it can lead to more biased sex ratios that can negatively impact sexual reproduction (Petry et al. 2016). According to a recent laboratory study on *M. inflexa*, photosynthetic pigments did not have sex-specific patterns due to light (Moore and McLetchie 2025). In contrast, a previous field study showed males occupying higher light conditions relative to females (Fuselier and McLetchie 2004). Collectively these findings suggest that light has sex-specific effects, but they may be subtle and trait dependent.

## Conclusion

Hydrated *M. inflexa* can survive very high temperatures that are lethal to many other plants. Spatial variation in thermotolerance is positively associated with light in both sexes but tends to be negatively associated with temperature, indicating that light is important in influencing heat resilience. Further, the thermotolerance-light association occurs more in males than in females. How and why this sex difference occurs is a fascinating area of research that requires further investigations. Overall, the findings of this study could lead to innovative research that unlocks the genetic secrets of the first land plant colonizers to mitigate heat stress and potentially improve resilience of heat-sensitive crop plants.

## Supplementary Material

plaf028_Supplementary_Data

## Data Availability

The data for this study are freely available at the following link: https://doi.org/10.6084/m9.figshare.29128913.v1.
