# Supplementary material for: Thriving in the tropics: spatial variation in heat resilience in the early diverging land plant, Marchantia inflexa
Source: AoB Plants. 2025 Jun 2;17(3):plaf028. doi: 10.1093/aobpla/plaf028 (PMC12206617; doi:10.1093/aobpla/plaf028)
Supplement: plaf028_Supplementary_Data [file plaf028_supplementary_data.pdf]

Supporting Information

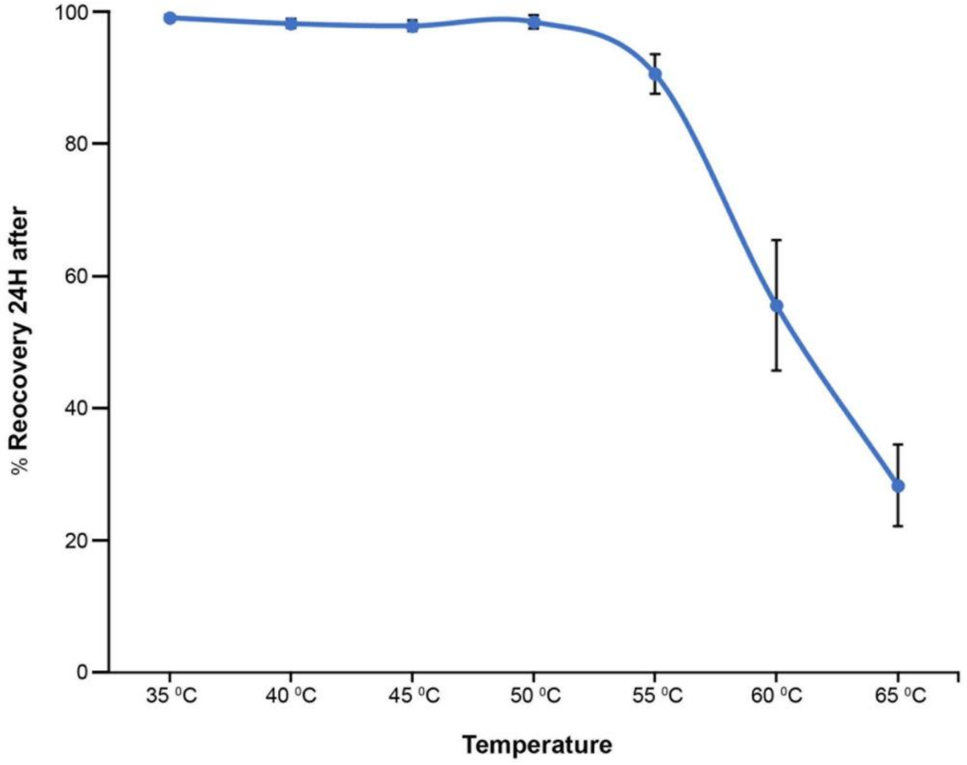

Figure S1: Recovery percentages of *Marchantia inflexa* thallus tips after 45 minutes exposure to a range of temperatures to determine the critical temperature to test plants in subsequent studies. Recovery F_v/F_m was taken 24 hours post the heat stress. Based on this preliminary study 55 ^{0}C was selected as a sublethal temperature that induces reduction in F_v/F_m without causing mortality, enabling assessment of differences in damage and recovery. Each data point represents the mean recovery from five replicates (n = 5) per temperature treatment.

Table S1: Habitat, light, daytime block, and their interaction predict recorded temperature on the warmest day. The table shows the effects, effect parameters and the p values of the repeated measures analysis. Streams and roads were analyzed separately. Effect parameters were used to predict the temperature for sites with no recorded temperatures.

Habitat	Fixed effect	Parameter estimate	Std error	t Ratio	Probability
Streams	Intercept	26.3581	0.1739	151.59	< 0.0001
	Instantaneous PPFD	0.0007	0.0003	2.55	0.0120
	Daytime block (1305h-1750h)	0.8470	0.1494	5.67	< 0.0001
	Instantaneous PPFD * Daytime block (1305h-1750h)	-0.0007	0.0003	-2.56	0.0117
Roads	Intercept	26.6283	0.4570	58.26	< 0.0001
	Instantaneous PPFD	0.0046	0.0005	9.08	< 0.0001
	Daytime block	3.5129	0.3481	10.09	< 0.0001
	Instantaneous PPFD * Daytime block	0.0027	0.0.0005	5.41	< 0.0001
